# What It Takes to Be a *Pseudomonas aeruginosa*? The Core Genome of the Opportunistic Pathogen Updated

**DOI:** 10.1371/journal.pone.0126468

**Published:** 2015-05-11

**Authors:** Benoît Valot, Christophe Guyeux, Julien Yves Rolland, Kamel Mazouzi, Xavier Bertrand, Didier Hocquet

**Affiliations:** 1 UMR CNRS 6249, Chrono-environnement, Université de Franche-Comté, Besançon, France; 2 UMR CNRS 6174, Institut FEMTO-ST, Département DISC, Université de Franche-Comté, Belfort, France; 3 UMR CNRS 6623, Laboratoire de Mathématiques de Besançon, Université de Franche-Comté, Besançon, France; 4 Mésocentre de calculs, Université de Franche-Comté, Besançon, France; 5 Laboratoire d’Hygiène Hospitalière, Centre Hospitalier Régional Universitaire, Besançon, France; The Scripps Research Institute and Sorrento Therapeutics, Inc., UNITED STATES

## Abstract

*Pseudomonas aeruginosa* is an opportunistic bacterial pathogen able to thrive in highly diverse ecological niches and to infect compromised patients. Its genome exhibits a mosaic structure composed of a core genome into which accessory genes are inserted *en bloc* at specific sites. The size and the content of the core genome are open for debate as their estimation depends on the set of genomes considered and the pipeline of gene detection and clustering. Here, we redefined the size and the content of the core genome of *P*. *aeruginosa* from fully re-analyzed genomes of 17 reference strains. After the optimization of gene detection and clustering parameters, the core genome was defined at 5,233 orthologs, which represented ~ 88% of the average genome. Extrapolation indicated that our panel was suitable to estimate the core genome that will remain constant even if new genomes are added. The core genome contained resistance determinants to the major antibiotic families as well as most metabolic, respiratory, and virulence genes. Although some virulence genes were accessory, they often related to conserved biological functions. Long-standing prophage elements were subjected to a genetic drift to eventually display a G+C content as higher as that of the core genome. This contrasts with the low G+C content of highly conserved ribosomal genes. The conservation of metabolic and respiratory genes could guarantee the ability of the species to thrive on a variety of carbon sources for energy in aerobiosis and anaerobiosis. Virtually all the strains, of environmental or clinical origin, have the complete toolkit to become resistant to the major antipseudomonal compounds and possess basic pathogenic mechanisms to infect humans. The knowledge of the genes shared by the majority of the *P*. *aeruginosa* isolates is a prerequisite for designing effective therapeutics to combat the wide variety of human infections.

## Introduction

*Pseudomonas aeruginosa* is a Gram-negative bacterium that causes significant mortality and morbidity among compromised patients, including those suffering from cystic fibrosis. The treatment of infected patients is complicated by the extraordinary capacity of this bacterium to develop resistance to almost all antibiotics, through the selection of mutations in chromosomal genes and the spread of horizontally acquired resistance [1]. Environmental and clinical isolates owe their extraordinary ability to thrive in many ecological niches and to harm many hosts to the conservation of metabolic and virulence genes in the genome of the species [2, 3]. Although *P*. *aeruginosa* has a non-clonal structure, a few sequence types (STs) called ‘high-risk clones’ are widely distributed and frequently encountered [4].

The genome of *P*. *aeruginosa* is large (> 6 Mbp) and exhibits a mosaic structure composed of a large core genome into which accessory genes are inserted *en bloc* at specific sites, called region of genomic plasticity (RGP) [5, 6]. While the diversity of genomic islands is well understood [6–8], there are still questions about the size of the core genome and its content. A thorough understanding of the networks of genes that are shared by the majority of the *P*. *aeruginosa* isolates is crucial for the design of effective therapeutics to combat the wide variety of human infections.

The size of the core genome has already been assessed *in silico* on small sets of clinical strains [6–8] or experimentally using DNA/DNA hybridization on a larger set of strains of various origins [3]. More recent studies have calculated the size of the core genome using a larger set of strains including various environmental isolates [2, 9]. *In silico* studies compared sets of genes annotated with different annotation pipelines. The discrepancies in the size of the predicted core genome of *P*. *aeruginosa* (from 4,455 to 5,316 genes) presumably rely (*i*) on the use of different annotation pipelines that annotate a given genome with inconsistencies such as misannotations or gene size errors [10], (*ii*) on the use of various set of strains, and (*iii*) on the definition of the core genes themselves (shared by all or nearly all the genomes). The progress and the decreasing cost of DNA sequencing techniques allow the researchers to access an increasing number of complete genomes. The size of the core genome is thought to further decrease with the addition of these new genomes.

We wanted here to determine the size and the content of the core genome of 17 strains of *P*. *aeruginosa* which gapless genomes were available in January 2014 on the NCBI database. The issue of annotation inconsistencies was circumvented by the re-annotation of all the genomes with the best performing annotation tool. We estimated the extent to which the addition of new genomes will further reduce the size of the core genome or expand that of the pan-genome. We discussed the conservation in the species of the genes implicated in the resistance to antibiotics, in the metabolism and respiration, and in the virulence.

## Materials and Methods

### Bacterial isolates and their genomic data

We downloaded from NCBI the nucleotidic sequences of the 17 gapless chromosomes of *P*. *aeruginosa* available in January 2014 (http://www.ncbi.nlm.nih.gov/nuccore/). The newest curated version of each isolate has been downloaded. The characteristics of the isolates are detailed in Table 1. Fourteen of the isolates were of clinical origin, of which 4 were isolated from cystic fibrosis patients (DK2, LESB58, PACS2, and RP73). Of note, LESB58 and LES431 are two UK isolates of the Liverpool epidemic strain and 19BR and 213BR are two Brazilian isolates that are nearly clonal [11]. Three isolates were isolated from the environment (M18, YL84, and MTB-1). When not available, we predicted the sequence type (ST) of the isolates from their whole genome sequence data with the MLST 1.7 tool [12].

**Table 1:** General features of the *P*. *aeruginosa* isolates used to define the core genome of the species.

*P*. *aeruginosa* isolates	Sequence type	Origin	GenBank accession	Genome size (Mb)	G+C%	Total gene cluster	Reference
PAO1	ST549	Clinical (Wound, Australia)	NC_002516.2	6.26	66.6	5,681	[13]
NCGM2.S1	ST235	Clinical (Urinary tract infection, Japan)	NC_017549.1	6.76	66.1	6,226	[14]
19BR	ST277	Clinical (Brazil)	AFXJ01000001.1	6.74	66.1	6,218	[15]
213BR	ST277	Clinical (Brazil)	AFXK01000001.1	6.72	66.1	6,184	[15]
B136-33	ST1024	Clinical (Diarrhea, China)	NC_020912.1	6.42	66.4	5,818	NBCI
DK2	ST386	Clinical (Cystic fibrosis, Denmark)	NC_018080.1	6.40	66.3	5,871	[16]
LESB58	ST146	Clinical (Cystic fibrosis, United Kingdom)	NC_011770.1	6.60	66.3	6,059	[17]
LES431	ST146	Clinical (United Kingdom)	NC_023066.1	6.55	66.3	6,006	NCBI
M18	ST1239	Environmental (Rhizosphere, China)	NC_017548.1	6.33	66.5	5,771	[18]
PA7	ST1195	Clinical (Argentina)	NC_009656.1	6.59	66.4	6,031	[8]
PACS2	ST1394	Clinical (Cystic fibrosis)	NZ_AAQW01000001.1	6.49	66.3	5,928	NCBI
RP73	ST198	Clinical (Cystic fibrosis, Germany)	NC_021577.1	6.34	66.5	5,804	[19]
UCBPP-PA14	ST253	Clinical (Burn wound)	NC_008463.1	6.54	66.3	5,908	[20]
YL84	CC169	Environmental (Compost)	CP007147.1	6.43	66.4	5,856	[21]
PA1	ST782	Clinical (Burn wound, China)	NC_022808.1	6.53	66.3	5,981	NCBI
SCV20265	ST299	Clinical (Cystic fibrosis, Germany)	NC_023149.1	6.73	66.3	6,190	[22]
MTB-1	Unknown^a^	Environmental (Contaminated soil, India)	NC_023019.1	6.58	66.2	6,000	[23]

^*a*^ The strain MTB-1 displayed the combination of alleles *acs-5*, *aro-8*, *gua-3*, *mut-5*, *nuo-1*, *pps-11*, and *trp-3* which corresponds to an unknown sequence type at the time of writing the manuscript.

### Gene prediction

Usual bioinformatics software tools (Prodigual, GeneMarkS, and Glimmer3) have been tested with the raw genome sequence of the reference strain PAO1 and confronted to the manually annotated sequences of the same strain [24–26]. Prodigal and Glimmer3 run in unsupervised mode, GeneMarkS can run either unsupervised with a heuristic approach or guided with a species-dependent configuration file.

### Gene clustering

A combination of a pairwise alignment score computation and a stochastic Markov cluster algorithm for graphs has emerged as the best method in terms of quality as well as speed of clustering. A proxy step has been added before the full score computation to speed up the process and to address the specific case of next to identical sequences. Both proxy and full score computations used the Sumatra software v1.0 (http://metabarcoding.org/sumatra) that couples a Needleman-Wunsch algorithm with a k-mer filter. The proxy step consisted in the pre-clustering of genes with > 98% of identity with a greedy incremental algorithm. The clustering has been completed using MCL software v12-135 with the removal of similarities <70% [27].

### Ortholog annotation

The most abundant nucleotidic sequence of each cluster (called “ortholog”) was functionally annotated with different databases. Clusters of orthologous groups (COGs) were determined with the Batch CD-Search tool [28]. Antibiotic resistance genes were identified by clustering (>98%) with the Antibiotic Resistance Database [29] and potential virulence factors investigated with the Virulence Factors Database [30].

### Computational and statistical analyses

Computations have been performed at the “Mesocentre de Calculs de Franche-comté”. All comparisons and filtering annotations were performed on R software (v3.2).

### Ethics statement

Not applicable.

## Results and Discussion

### Validation of the method

We compared the gene detection by three usual bioinformatics tools (Prodigual, GeneMarkS, and Glimmer3) with the 5,542 manually annotated genes of the reference strain PAO1 [24–26]. Prodigual provided the best result compared to GeneMarkS and to Glimmer3 (Table 2). Hence, Prodigual predicted 90.9% of the annotated genes and found 238 false positive genes. In comparison, the performances for GeneMarkS (78.2% of accuracy and 373 false positive genes) and Glimmer3 (84.7% of accuracy and 475 false positive genes) were much lower. We therefore submitted all genomes to an *ab initio* gene prediction using Prodigal software. Gene and protein clustering can be handle with a rapid algorithm using a greedy incremental approach [31] or by graph clustering on similarity matrix [27, 32]. Here, we performed a hybrid approach. Genes were first rapidly clustered using the Sumaclust program that uses the same clustering algorithm as CD-HIT [31]. Then the seed of each cluster served as a proxy for pairwise comparison and the whole matrix went through a classification process with the MCL clustering program. In order to validate our clustering model which is a crucial step for the determination of core and pan-genomes, we assessed the influence of the similarity cutoff on their size. The size of the core and the pan genomes was stable with gene similarity cutoffs between 65 and 75% with a minimal influence of the inflation parameter (S1 Fig). Cutoff was set at 70% because it maximizes the number of orthologs within the core genome with only one gene per genome, therefore reducing the risk of overclustering. Unlike pipelines that use local similarity search tool (*i*.*e*., BLAST), we compute a global similarity value to keep the raw values before graph resolution.

**Table 2:** Comparison of the gene annotation by Prodigual, GeneMarkS, and Glimmer3 with the annotated sequences of the reference strain PAO1.

	GeneMarkS	Prodigual	Glimmer3
**Identical** ^**a**^	4,333 (78.2%)	5,039 (90.9%)	4,694 (84.7%)
**Partial** ^**b**^	1142	468	763
**False positive** ^**c**^	373	238	475

The values indicate the numbers of predicted genes falling into each category. The percentages indicate the proportion of the 5,542 annotated genes of PAO1 correctly annotated (100% identical).

^a^Predicted gene is 100% identical with a reference gene.

^b^Predicted gene is ≥ 50% identical with a reference gene.

^c^Predicted gene is < 50% identical with a reference gene.

### Core genome limits

**Fig 1:**
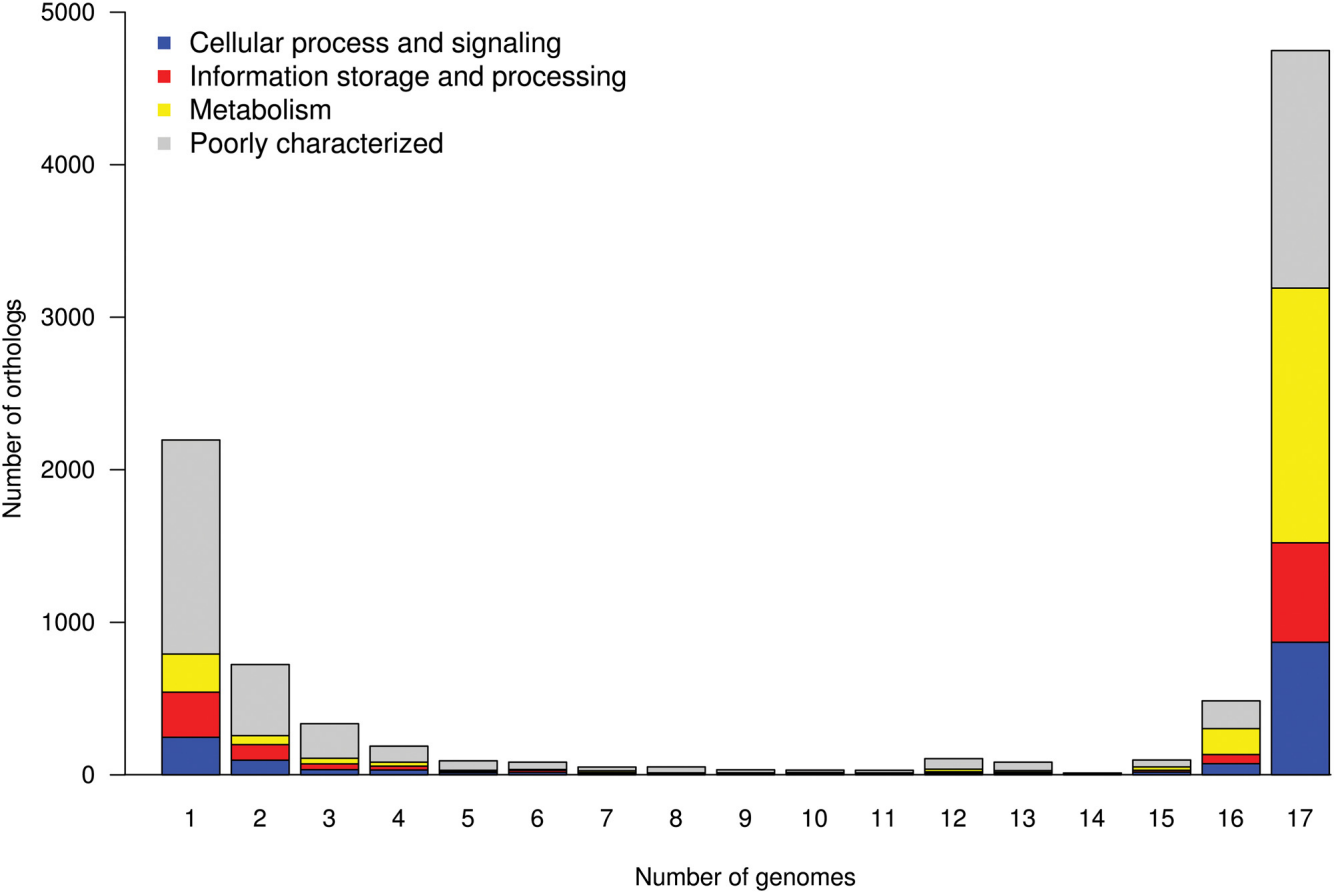
Distribution of orthologs in genomes. Number of orthologs as a function of the number of genomes they are in, broken down by super-functional categories using COG database.

**Fig 2:**
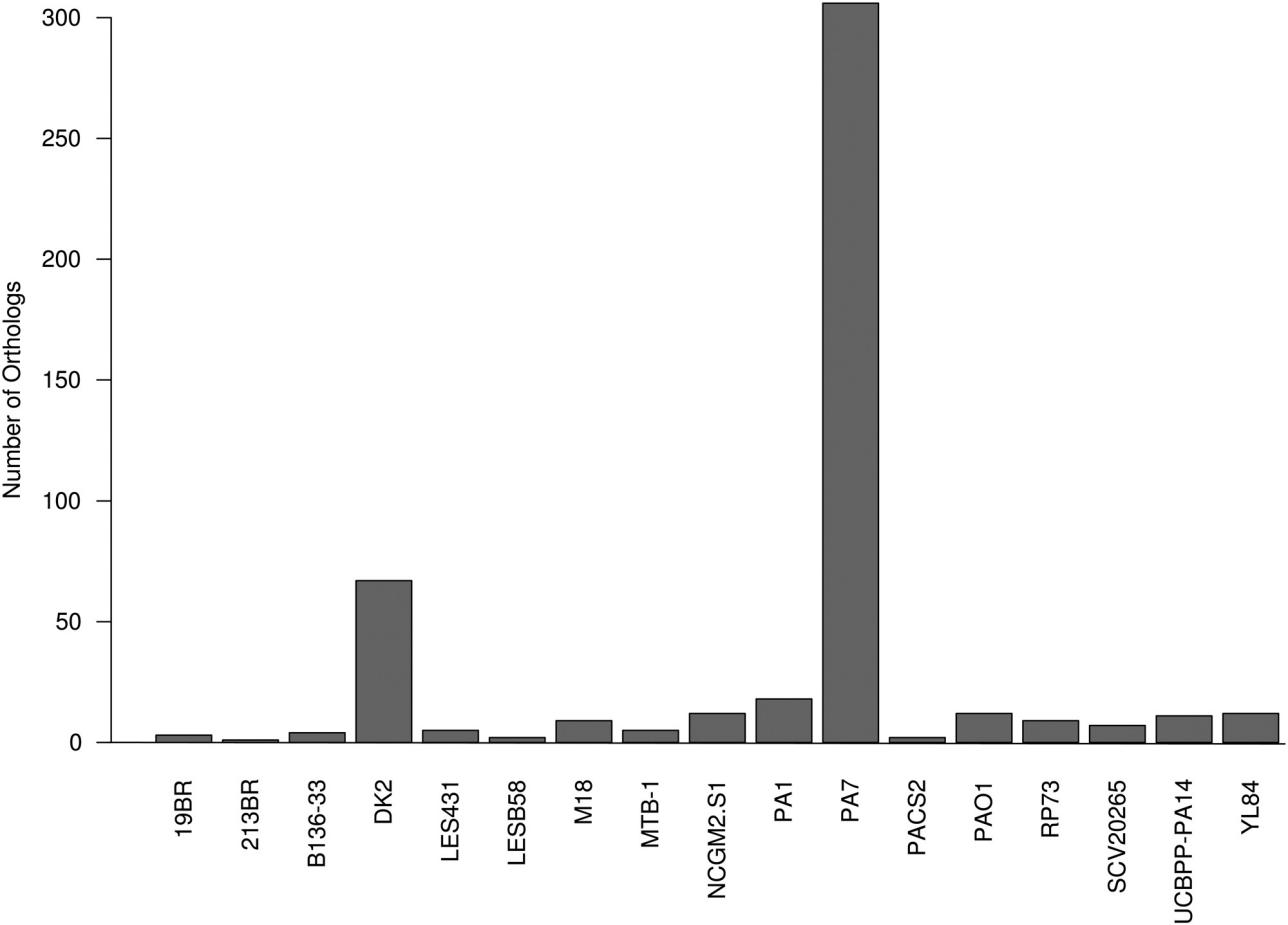
Isolates of origin of the 485 orthologs shared by 16 out of the 17 tested genomes.

The clustering of the genes of the 17 genomes retrieved 9,344 orthologs that we annotated using COGs (S1 Table). The distribution of these orthologs is clearly uneven with the great majority (8,151/9,344; 87.2%) of the orthologs either present in ≤ 2 genomes or in ≥ 16 genomes (Fig 1). Interestingly, 485 orthologs are found in all but one genome (that is, 16). The distribution of the putative super-functional COG categories of the orthologs found in 16 and 17 genomes was similar. We sought for the origin of the 485 orthologs absent from only one genome. Clearly, the distribution was not random with the two isolates PA7 and DK2 representing 373 out of 485 orthologs (Fig 2). The phylogenetic trees built by Grosso-Becerra *et al*. show that PA7, although distant from the bulk of the other *P*. *aeruginosa* strains, is clearly in the branch of the species *P*. *aeruginosa* [2]. The isolate DK2 was isolated from a chronically infected patient suffering from cystic fibrosis, and characterized by a large deletion in the core genome [16]. The core genome could be restricted to the 4,748 genes shared by the entire genome collection. However, to take into account phylogenetic outliers and host-adapted isolates, we included in the core genome the genes present in *n*-1 genomes (that is, 16). Consequently, the accessory genome was defined thereafter as the set of genes shared by < 16 genomes.

### Core and pan-genome size of *P*. *aeruginosa*

**Fig 3:**
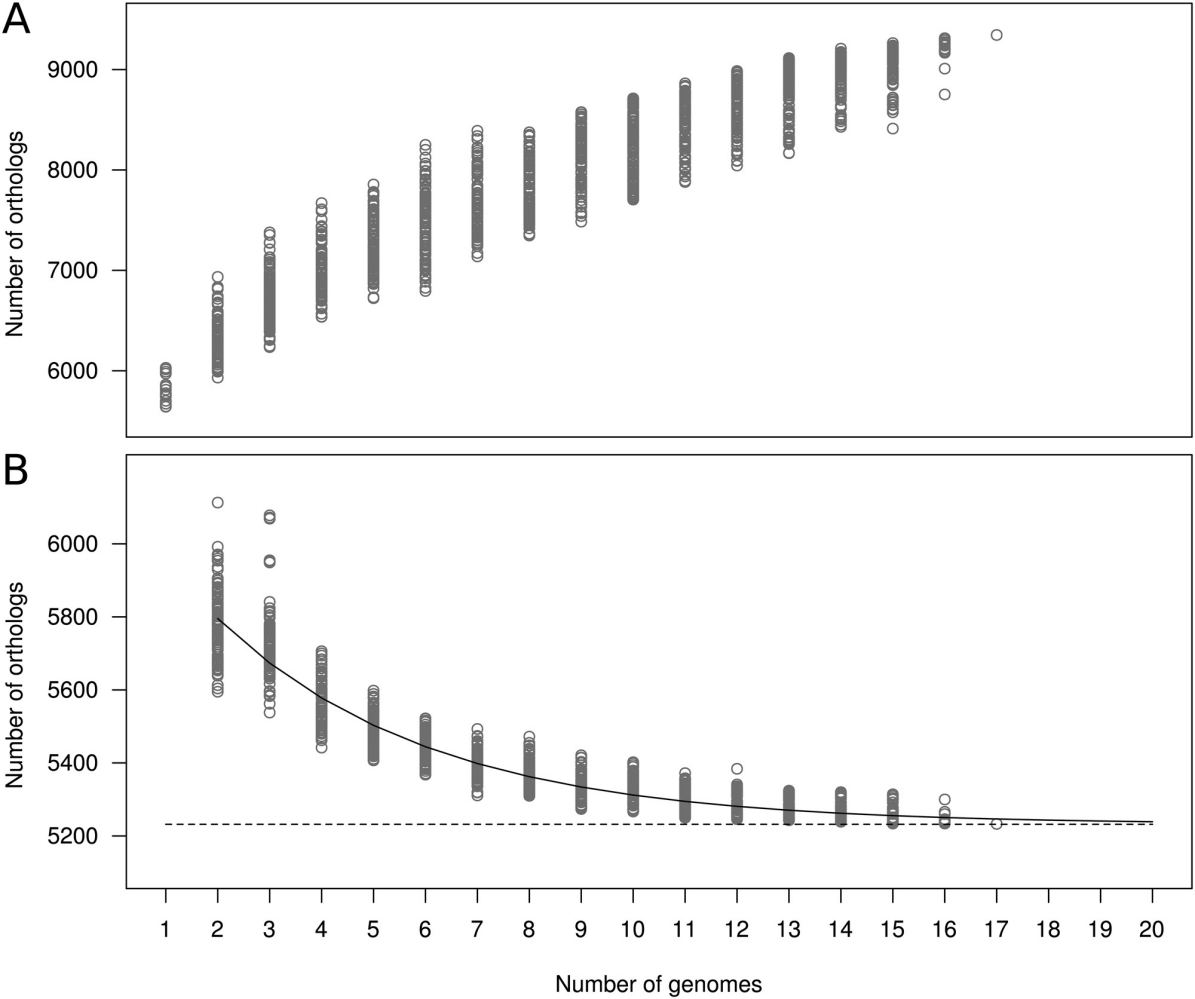
*Pseudomonas aeruginosa* pan (A) and core (B) genome size according to the number of sequenced genomes (*n*). The amount of shared orthologs is plotted as a function of the number of genomes sequentially added. One hundred clusterings for each *n* value (except for *n* = 1 and 17) were performed by random selection of *n* genomes (Table 1). On the panel B, the continuous curve represents the least-squares fit of the function Fc = a.*e* ^*-b*.*x+c*^ + d to data. The best fit was obtained with correlation *r* ^*2*^ = 0.913 for a = 1.03, b = 0.24, c = 4.48, and d = 5,232 (95% confidence interval for d = 5,219–5,245). The extrapolated *P*. *aeruginosa* core genome minimal size is shown as a dashed line.

Using a clustering approach, we found that the average *P*. *aeruginosa* genome contained 5,972 genes, the pan-genome 9,344 genes (Fig 3A), and the core genome 5,233 genes (Fig 3B). Using a smaller set of genomes (*i*.*e*., ≤ 5), other authors found comparable results [6–8]. This suggested that further sampling of *P*. *aeruginosa* was unlikely to significantly reduce the size of the core genome. We tested this hypothesis by estimating the variation of the core and pan-genome using random sets of genomes (Fig 3B). As expected, the number of shared genes decreased along with the addition of each new genome. Nevertheless, the extrapolation of the curve indicates that the core genome reaches a minimum of 5,232 (95% confidence interval = 5,219–5,245). This value is in line with the size of the core genome calculated above and will remain relatively constant, even if more genomes are added. Thus, the core genome is highly conserved and represents ca. 88% of the average genome. In other words, the full sequencing of a *P*. *aeruginosa* strain allows the observation of *ca*. two-thirds of the calculated pan-genome. This implies that most fundamental functions can be studied with a model strain and extrapolated to the species.

*P*. *aeruginosa* has a larger genome than those of the two other major nosocomial pathogens *Escherichia coli* and *Staphylococcus aureus* that have an average size of 4,721 and 3,118 genes, respectively [33, 34]. The ubiquity of *P*. *aeruginosa* relies on its metabolic versatility and on the large range of its hosts. These two properties are the consequence of the large genome of the species. Besides, the small size of the accessory genome of *P*. *aeruginosa* reflects the absence of strain clustering during evolution and the minimal adaptation to environmental niches (with the exception of chronic infections).

The disparity between the very large pan-genome of *E*. *coli* (17,838 genes) and its relatively small core genome (1,976 genes) reflects the different interaction modes with hosts, from commensal to highly pathogenic [33]. This contrasts with the clonal and pathogenic *S*. *aureus* which pan-genome size (3,221 genes) is very close to that of the average genome size (3,118 genes)[34].

### Conservation of the metabolic and respiratory genes

**Fig 4:**
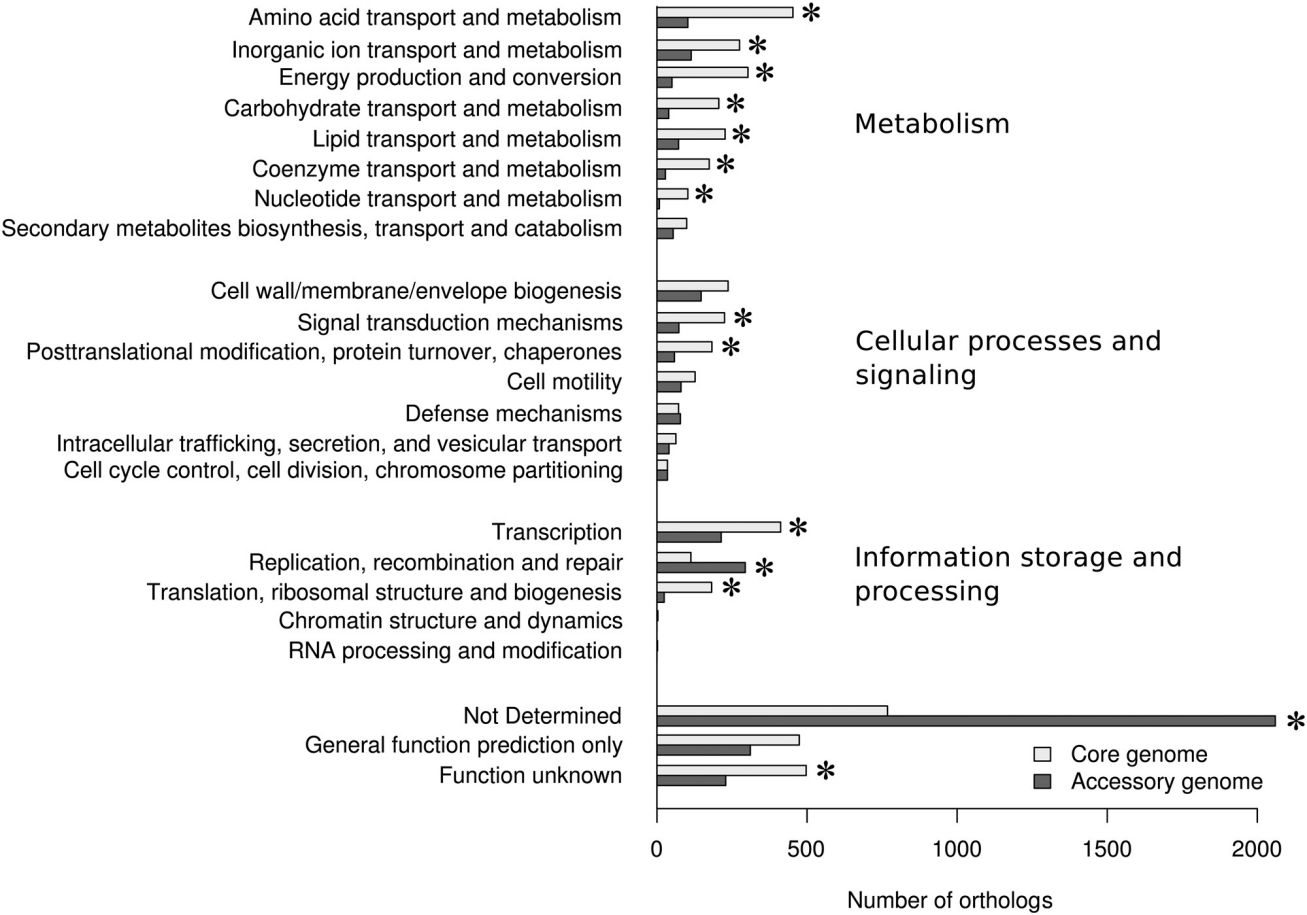
Distribution of core and accessory genes sorted by COG functional categories. Orthologs without any COG annotation were sorted as ‘not determined’. The asterisks indicate statistical significance at *p*-value < 0.01.

The genes involved in the metabolism are mostly conserved and significantly enriched in the core genome (except genes involved in the secondary metabolites biosynthesis) (Fig 4). More precisely, 1,840 out of the 2,304 metabolic genes were found in core genome (79.9%). We also found that the genes involved in aerobic and anaerobic respiratory metabolism were in the core genome. Hence, genes that encode (*i*) the five terminal oxidases for aerobic respiration, (*ii*) the denitrification enzymes (*nar*, *nap* clusters for nitrate reductases; *nir* cluster for nitrite reductase; *nor* cluster for NO reductase, and *nos* cluster for N_2_O reductase), and (*iii*) the enzymes for anaerobic fermentation (*arc* cluster) were present in the 17 studied genomes. The conservation of metabolic and respiratory genes guarantees the ability of the species to thrive on a variety of carbon sources for energy in both aerobiosis and anaerobiosis. Similarly, genes involved in transcription and translation are enriched in the core genome. As expected, accessory genome contained many genes that are not determined, either because they are understudied or because they are pseudogenes. Interestingly, accessory genome was also significantly enriched with genes involved in replication, recombination, and repair (Fig 4), among which genes that encode integrases, recombinases, and transposases, responsible for DNA mobility.

### Core and accessory signature

**Fig 5:**
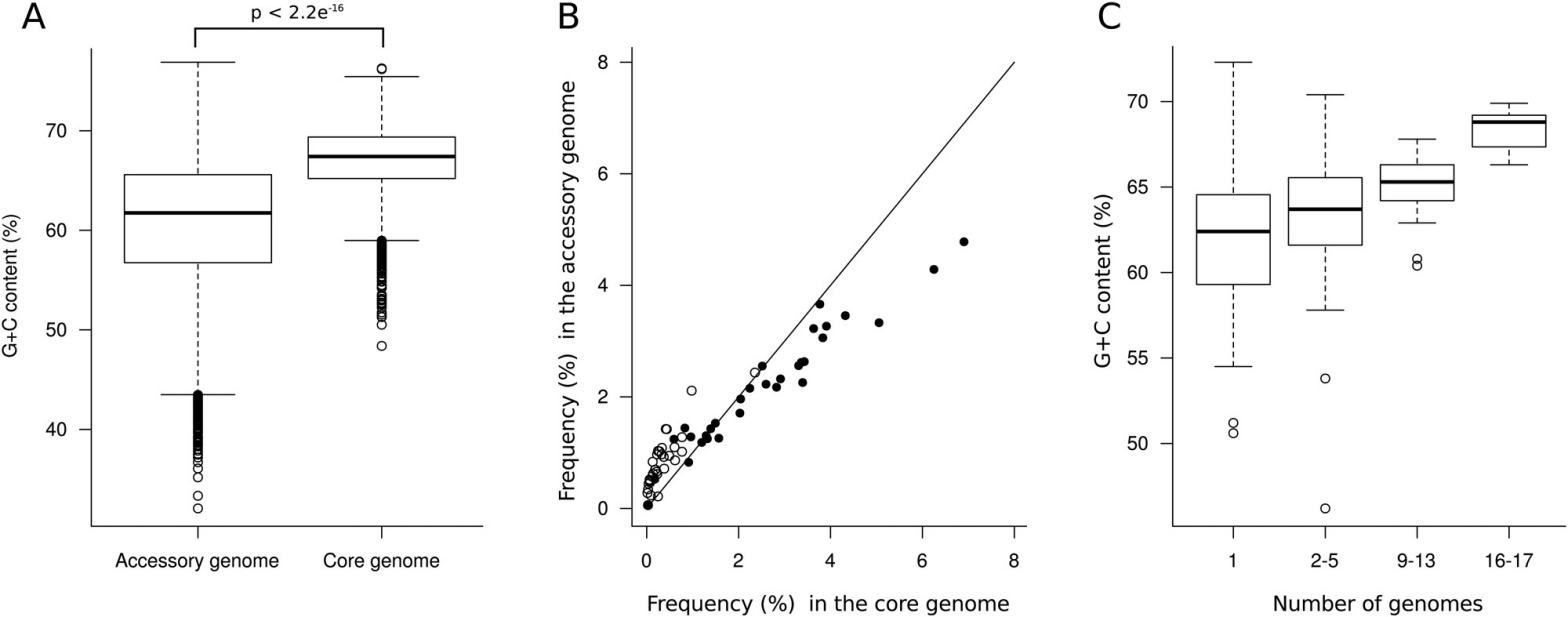
Comparison of the G+C content, codon frequency between core and accessory genome and genetic drift of prophage elements. (A) Comparison of the G+C content between core and accessory genome. The box plots represent the first and third quartiles for each *n* value, the solid line indicated the median (61.7% for accessory genes and 67.1% for core genes; two-sided Student's t-test), the whiskers indicate the maximums and the open dots indicate the outliers. (B) Codon frequency (%) compared between core and accessory genome. Open circles indicate codons ending with A or T, black circles indicate codons ending with G or C. (C) G+C content of prophage elements according to the number of genomes they are in. The solid line indicated the median (62.4% for unique prophage elements and 68.8% for core prophage elements). The whiskers indicate the maximums and the open dots indicate the outliers.

*P*. *aeruginosa* genome is characterized by a high G+C content [13]. Genes acquired from other species (typically belonging to the accessory genome) generally have a lower G+C content than that of the *P*. *aeruginosa* core genome [5]. We confirm here that the accessory genome has a lower G+C content (61.7%) than that of the core genome (67.1%) (Fig 5A). Grocock and Sharp evaluated the frequency of G+C at the third synonymously variable coding position at 83% in *P*. *aeruginosa* PAO1 [35]. This value was highly variable and was low in putatively foreign genes. Here, we compared the codon frequency between core and accessory genome (Fig 5B). A high correlation was found and reflected the codon bias of *P*. *aeruginosa* genome. However, the codons ending with G+C were more frequent in core genes than in the accessory genes, while codons ending with A+T were more frequent in the accessory genome than in the core genome.

Once integrated into a bacterial chromosome, foreign DNA experiences the same pressures as the rest of the genome and may lose over time the sequence compositional differences that distinguished it from the core genome [5]. To test this hypothesis in *P*. *aeruginosa*, we assessed the median G+C content of prophage elements according to their presence in *n* tested genomes. We found that unique prophage elements typically displayed the signature of accessory genes (median GC%: 62.4%) while long-standing elements were subjected to a genetic drift in *P*. *aeruginosa* and reach similar G+C content as core genes (68.8%)(Fig 5C). In contrast, genes encoding housekeeping functions are not always affected by this drift. Hence, many of the core genes encoding the translational apparatus (36 out of the 57 ribosomal proteins L and S) displayed an atypical signature (G+C content ≤ 60%). Weinel *et al*. also observed this pattern in the genome of the metabolically versatile *Pseudomonas putida* [36]. By taking up their idea, highly conserved ribosomal genes of *P*. *aeruginosa* could have evolved with a less stringent bias towards GC-rich codons and hence prefer codons other than the typical *P*. *aeruginosa* genes. In other words, the ribosomal proteins do not compete with typical species proteins for the same tRNA molecules during translation, and that the utilization of separate tRNA pools could facilitate the metabolic versatility of *P*. *aeruginosa* [36].

### Antibiotic resistance genes in the core genome

*P*. *aeruginosa* has a formidable capacity to become resistant to nearly all the antibiotics of the market [37]. Although resistance determinants can be acquired by horizontal transfer (especially to β-lactams and aminoglycosides), *P*. *aeruginosa* strains may readily adapt themselves to the antibiotic pressure via chromosomal mutations and do not necessarily require the transfer of foreign DNA. We found here that the gene encoding the intrinsic cephalosporinase AmpC, those encoding the drug efflux pumps MexAB-OprM, MexCD-OprJ, MexXY, MexEF-OprN, those encoding the fluoroquinolone-targeted DNA gyrase and topoisomerase IV (*gyrA*, *gyrB*, *parC*, and *parE*) were in the core genome. The maintenance of the gene encoding the AmpC cephalosporinase in all the studied strains can be due to the presence of β-lactams in all the niches, but also to the morphological role of this enzyme [38]. The extensive conservation of the drug efflux pumps in all the strains, regardless of their origin, clinical or environmental, suggests a selection for their maintenance throughout the evolution. This could be related to the involvement of efflux pumps in the survival of *P*. *aeruginosa* in their ecological niche, as demonstrated for MexAB-OprM [39]. Hence, it is worth noting that environmental strains of *P*. *aeruginosa*, that are usually susceptible to antipseudomonals, have the complete toolkit to become resistant to all these compounds, via mutations (*i*) of fluoroquinolone targets or (*ii*) in the gene coding for the porin OprD, or (*iii*) of regulators leading to the overproduction of the AmpC cephalosporinase or efflux pumps.

### Accessory virulence genes of core functions

**Fig 6:**
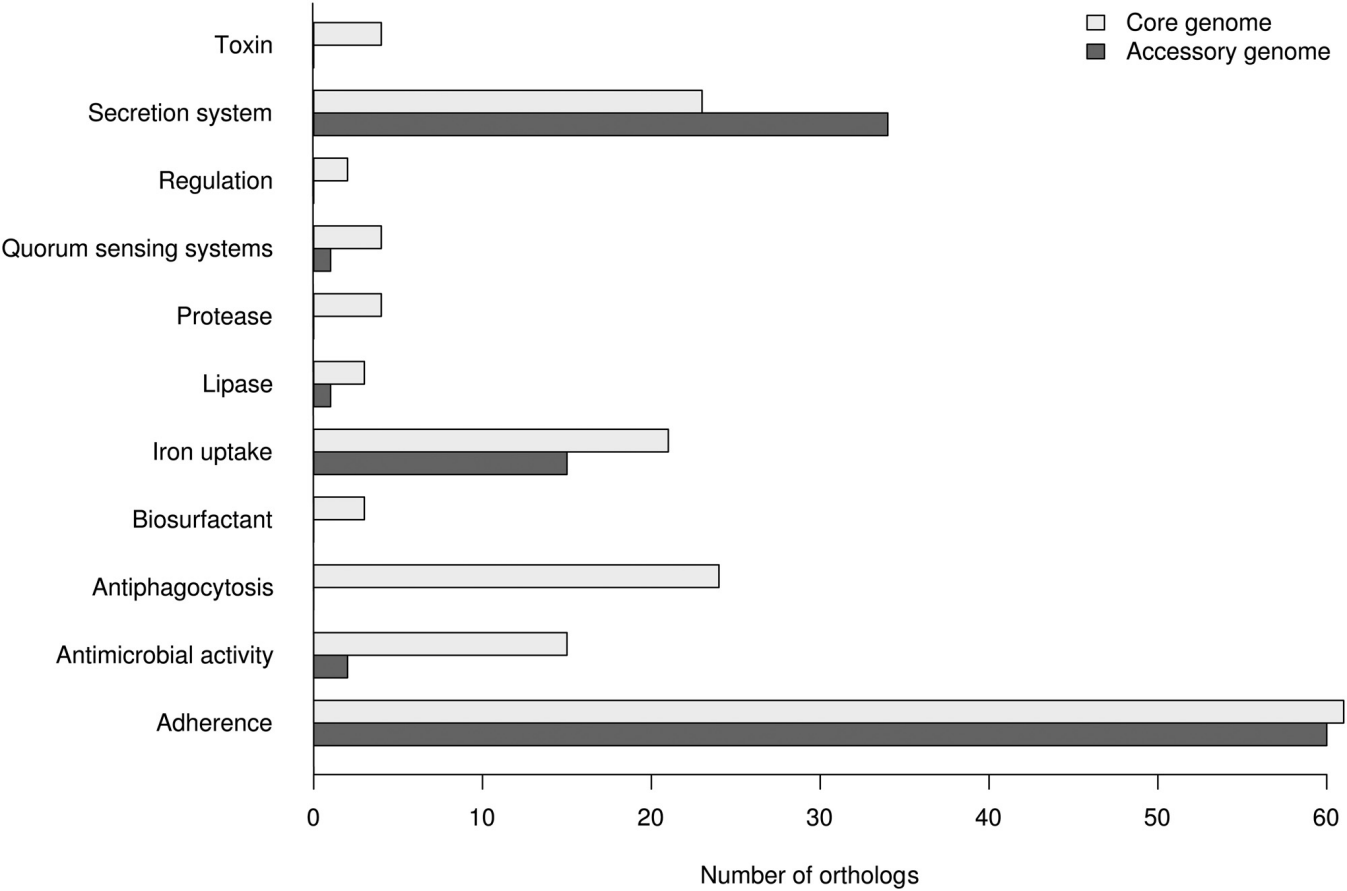
Distribution of virulence genes in core and accessory genomes of *P*. *aeruginosa*. The virulence genes were detected with the Virulence Factors Database [30] and their distribution were plotted as a function of the core and accessory genome.

Less than two third (164 out of 277) virulence genes are found in the core genome (Fig 6). This contrasts with the extensive conservation of virulence determinants among strains [3, 20]. The genes coding for components of the biosynthesis of O-antigens of the lipopolysaccharide (e.g., *wcaG*, *wzzB*, *wec* genes, *rfbX*, *rfaG*, *rfe*) accounted for the vast majority of accessory genes and present in low number of genomes (1 to 5). This sequence diversity in O-antigen biosynthesis genes is at the origin of the O-serotype of the strains [40].

Several types of type IV pili have been described [41]. Some *pil* genes are accessory and determine the type IV pili allele. Hence, environmental strains M18, MTB-1, YL84 and cystic fibrosis strains LESB58 and LESB431 were of type I (with *tfpO*) whereas PAO1 was of type II and PA7 strain of type IV. Interestingly, the CF-strains DK2 and RP73, and the reference strain PA14 shared an accessory gene downstream of *pilA* (with >98% of identity) of a not yet described type.

The genes *exoT*, *exoY*, *exoU*, and *exoS* encode four type III secreted proteins [42]. *ExoT* and *exoY* are core genes although not in the outlier PA7. The genes *exoU* and *exoS* are mutually exclusive with *exoU* found in the strains B136-33, MTB-1, NCGM.S2 and PA14 and *exoS* found in the other studied strains. As noticed before, PA7 has neither *exoS* nor *exoU* [8].

We also observed variations in the sequences and the combination of the genes in the pyoverdine locus involved in iron uptake [43]. Although some *pvd* genes belonged to the core genome (*pvdAGHLMNOPQS*), genes encoding the siderophore pyoverdine and the receptor for ferripyoverdine (e.g., *pvdD*, *pvdE*, *pvdF*, *pvdI*, *pvdJ*, *pvdY*, and *fpvA*) are shared by only a subset of strains (see S1 Table).

Strains of *P*. *aeruginosa* either produced flagellin of a-type or b-type [44]. In our series, we identified the 4 accessory genes (corresponding to the cluster *pa1088-pa1091* in PAO1) involved in the biosynthesis of a-type flagellin in 7 genomes (19BR, 213BR, DK2, LESB58, LESB431, PA14, and PAO1). In contrast, the strains B136-33, M18, MTB-1, NCGM2.S1, PA1, PA7, PACS2, RP73, SCV20265, and YL84 harbored 11 other flagellar glycosylation genes necessary for the production of b-type flagellin [44]. A set of secretion system genes was accessory (Fig 6). These genes were absent only from the 2 isolates PA7 and RP73, which lacked a 25-gene cluster *pscQ-pscL*. It corresponds to the cluster *pa1694-pa1725* in PAO1, and encodes the type III secretion system that enables the injection of toxins into host cells [8].

The genes involved in determining the O-serotype, type IV pilus, siderophore production and flagellin type can be accessory genes because of either their sequence divergence or their presence in a subset of strains. However, the biological functions resulting from these gene clusters are preserved throughout the evolution and it is arguable that they are really core functions [17]. Hence, nearly all the virulence determinants are maintained throughout evolutionary pressure in strains regardless of the source (environmental or clinical). Although little evidence exists, the selection and maintenance of pathogenicity may occur constantly in the environment where *P*. *aeruginosa* potentially encounters a large range of hosts such as nematodes, insects, plants, and amoeba [20]. This implies that virtually all the strains possess basic pathogenic mechanisms to infect humans [3].

### Limitations of the approach

To focus on high-quality data, we only extracted gapless chromosomes available on NCBI. This collection is somehow limited (*n* = 17) when compared to the 289 genome assembly and annotation reports for the species at the time of this writing. Of note, the collection included only 3 isolates from 'high-risk clones’ (ST235 and ST277) (Table 1).

The extrapolation from ortholog clustering based on nucleotidic sequences to the functional content (*e*.*g*., virulence, metabolism, resistance) is uncertain. For example, although the gene encoding the AmpC cephalosporinase regulator AmpR was retrieved in all the studied strains and therefore part of the core genome, a thorough examination of the nucleotidic sequences of *ampR* in multi-drug resistant isolates revealed 2 non-silent mutations in the strain NCGM2.S1 and 13 non-silent mutations and two 3-bp insertions in the strain PA7, that probably impair the AmpR function. Some strains can also harbor mutated regulators (*e*.*g*., LasR for strains PA7 and NCGM2.S1, MexZ for strains SCV20265, PA7, DK2, and PA7) with impaired functions. This bias presumably artificially inflates the size of the core genome. In contrast, the adaptation of *P*. *aeruginosa* to chronic infection (*e*.*g*., in the lungs of chronically-infected cystic fibrosis patients) is sometimes associated with the loss of massive fragments of chromosome [45]. Hence, Ernst *et al*. described host-adapted isolates that had lost ‘*en bloc*’ more than 100 genes designed here as core genes. The inclusion of such particular isolates from chronically infected patients, adapted for the growth in their niche but presumably no fit enough for surviving out of their hosts, would artificially reduce the size of the core genome [46]. It gives the rationale of our less stringent definition of core genome, that includes *n*-1 genomes and that takes into accounts chronically adapted isolates (DK2) or outliers (PA7).

## Conclusions

Despite the low genomic diversity between strains and the conservation of virulence genes, some strains called ‘high-risk clones’ are more prone to disseminate. This feature is certainly related, but could not be totally attributed, to their resistance to antibiotics [47]. The study of more ‘high-risk clones’ could identify particular genes responsible for their spread in clinical settings. The high number of conserved genes in the core genome of *P*. *aeruginosa* allows the metabolic versatility of the species for various environmental niches, its infectious capability towards a large set of hosts, and its capacity to become readily resistant to antibiotics. The knowledge of the genes shared by the majority of the *P*. *aeruginosa* isolates would help for the design of effective therapeutics to combat the wide variety of human infections.

## Supporting Information

S1 Fig Influence of the clustering parameters on the size of the core and the pan genome of *P*. *aeruginosa*. The number of orthologs in the core (gray) and the pan (black) genome according to the identity threshold. The influence of the inflation parameter (from 2 to 4) was negligible for the clustering (data not shown). We therefore set the inflation at 3.
(DOCX)

S1 Table Annotation of the 9,344 orthologs found in the genomes of 17 *P*. *aeruginosa* isolates. Each ortholog is annotated with Prodigual using COG and Uniprot databases. The genomes and the number of genomes (from 1 to 17) in which the orthologs are found, and the numbers of copies in each genome are given. The G+C content and PAO1 gene numbering is given for each ortholog, when available. The signification of the COG abbreviations is detailed in the second worksheet.
(XLS)
