# Supplementary figures and images for: What It Takes to Be a Pseudomonas aeruginosa? The Core Genome of the Opportunistic Pathogen Updated
Source: PLoS One. 2015 May 11;10(5):e0126468. doi: 10.1371/journal.pone.0126468 (PMC4427113; doi:10.1371/journal.pone.0126468)

**S1 Fig.**


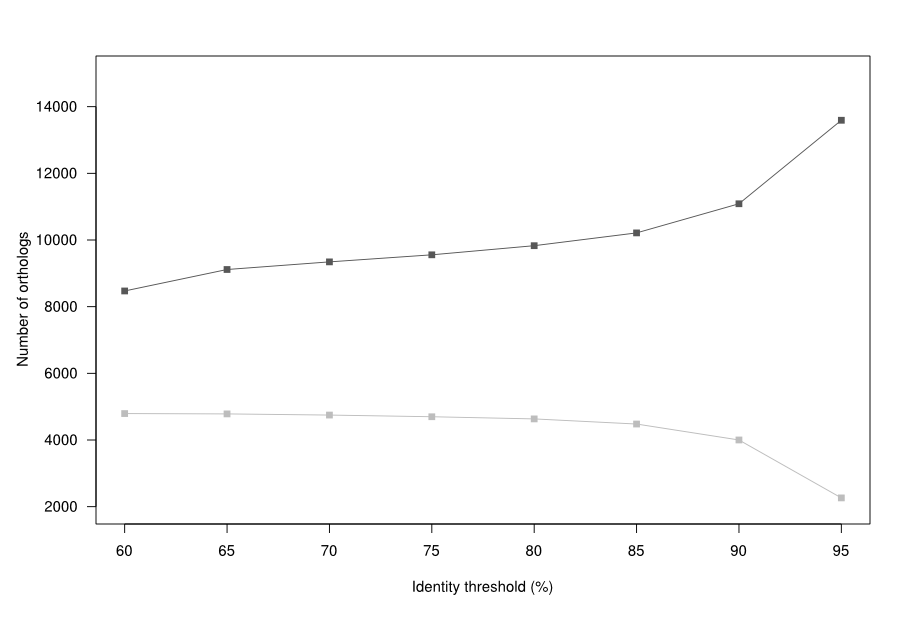

Supplement: S1 Fig — The number of orthologs in the core (gray) and the pan (black) genome according to the identity threshold. The influence of the inflation parameter (from 2 to 4) was negligible for the clustering (data not shown). We therefore set the inflation at 3. (DOCX) [file pone.0126468.s001.docx]
